# Who is pausing immunosuppressive medication for COVID-19 vaccination? Results of an exploratory observational trial

**DOI:** 10.1186/s40001-022-00727-7

**Published:** 2022-06-22

**Authors:** Dominik Schröder, Stephanie Heinemann, Gloria Heesen, Frank Klawonn, Marie Mikuteit, Jacqueline Niewolik, Sandra Steffens, Georg Behrens, Alexandra Jablonka, Frank Müller

**Affiliations:** 1 Department of General Practice, University Medical Center Goettingen, Goettingen, Germany; 2 Department of Computer Science, Ostfalia University of Applied Sciences, Wolfenbuettel, Germany; 3 German Center for Infection Research (DZIF), Partner Site Hannover, Braunschweig, Germany; 4 Department of Rheumatology and Immunology, Hannover Medical School, Hannover, Germany; 5 Biostatistics Group, Helmholtz Centre for Infection Research, Braunschweig, Germany

**Keywords:** Immunosuppression, SARS-CoV-2, COVID-19, Immunization, Immunogenicity, Drug compliance, Drug adherence

## Abstract

**Background:**

The influence of immunosuppressive therapy on immunogenicity after COVID-19 vaccination remains unclear. This study surveys patients who receive immunosuppressive therapy about whether or not they paused their immunosuppressive medication while receiving SARS-CoV-2 vaccination.

**Methods:**

In this prospective observational study, immunosuppressed participants were asked by phone and email about their medication before and during vaccination and who—if anyone—advised them to pause their medication. In addition, a baseline paper-based questionnaire contributes general characteristics regarding age, gender, immunosuppressive medication(s) and the chronic disease(s) requiring immunosuppressive therapy.

**Results:**

Of 207 surveyed participants, 59 persons (28.5%) paused their immunosuppressive medication before/during vaccination. Persons with rheumatic conditions and women were significantly more likely to pause immunosuppressive therapy than others. Over half of those who paused their medication reported receiving a recommendation from their specialist and 22.0% (13 of 59) decided to pause medication themselves without consulting a physician in advance.

**Conclusions:**

Besides lack of evidence, many immunosuppressed individuals and their treating physicians choose to pause medication before COVID-19 vaccination and accepting the risk of worsening their underlying disease.

*Trial registration*: DRKS00023972, registered 12/30/2020.

## Introduction

Patients and their treating physicians face uncertainty in deciding whether to pause immunosuppressive therapy prior to and after vaccination for COVID-19. One argument in favor of pausing therapy would be to possibly obtain a more solid immune response and better immune protection against SARS-CoV-2 infection. In contrast, there is a risk of a relapse of the underlying disease, possibly with serious consequences, such as organ rejection in transplant patients. So far, it is still not sufficiently clear whether and which immunosuppressive therapy reduces the vaccination response in COVID-19 vaccinations and, conversely, whether a short-term pause can counteract this. For other vaccinations, such as influenza vaccination, better immunogenicity was found when immunosuppressants, such as methotrexate, were paused [1]. However, these results do not readily translate to mRNA-based vaccines.

First studies show that methotrexate has an inhibitory effect on humoral immune responses to the COVID-19 vaccine BNT162b2 (Pfizer BioNTech, single shot), whereas cellular responses are preserved [2, 3]. Similar effects can be assumed for corticosteroids and mycophenolate medication [4–6].

In a case report of an immunosuppressed non-responder vaccinated after two doses of BNT162b2, successful antibody detection was achieved after two additional vaccinations with pause of the existing medication (mycophenalate and prednisone) [7].

Organ transplant patients are generally not recommended to discontinue immunosuppression because of the serious consequences of organ rejection [8], however, some authors discuss pausing immunosuppressive medications, if the risk of long time worsening of a condition is minimal, e.g., for atopic dermatitis [2].

In the face of this unclear recommendation and information situation, patients may tend to pause their immunosuppressive medication independently and without prior risk education. With the present study, we would like to describe which patient groups tend to pause their medication and who advises them to pause.

## Methods

### Study design and participants

The study is part of the CoCo Immune Study [9]. This observational study aims to study immune response, social participation and attitudes towards vaccination in people receiving a vaccination against SARS-CoV-2 that carry a great risk for a severe SARS-CoV-2 disease course. This paper reports on the subgroup of participants taking regularly immunosuppressive medication.

Participants, at least 18 years old, with regular intake of an immunosuppressive medication (defined as any drug therapy of the Anatomical Therapeutic Chemical (ATC) group L04 or a systemic corticoid therapy with a prednisone equivalent of ≥ 2.5 mg/day at enrollment) and full immunization against SARS CoV-2 not have occurred more than 30 days prior to study enrollment (counted from the first vaccination for Johnson & Johnson vaccine or the second vaccination for all other vaccinations) were recruited in this cohort.

Potential participants were informed about the study by newspaper announcements, homepage, social media posts, posters at vaccination centers, local general practices and clinics for patients requiring immunosuppressive therapy throughout the Northern German region of Lower Saxony.

Study participants were excluded from the analyses if they did not state their immunosuppressive medication or underlying condition.

No treatment or counseling was provided as part of the study. No intervention took place. The study participants were thus treated exclusively by physicians outside our own clinics and did not receive any compensation for their participation. Recruitment was based on a pragmatic sample and was independent of the diseases underlying the immunosuppressive medication (real-life sample).

### Data collection and management

At enrollment, participants completed a paper-based self-reported questionnaire on sociodemographic characteristics (age, gender, education level), medical characteristics (diseases, medication therapy, symptoms of depression and anxiety assessed by PHQ-4 questionnaire [10, 11]) and COVID-19 specific characteristics (previous SARS-CoV-2 infection, vaccine used for immunization). Between 07/21/2021 and 08/21/2021, study nurses contacted participants by phone or email, asking if they have paused their immunosuppressive medication before or after first and/or second SARS-CoV-2 immunization. In addition, participants who paused their immunosuppressive medication were asked who recommended pausing their medication. Three additional phone calls, or a reminder email, were made if contact attempts were unsuccessful. Responses via email were received until 08/09/21. Data were entered into the EvaSys digital survey system (EvaSys GmbH, Lüneburg, Germany) and exported from there into SPSS (.sav) data format

### Statistical analyses

Sociodemographic and medical characteristics were compared between the participants who gave information about pausing their immunosuppressive medication and participants who were not reached in the follow-up. Participants who paused their medication (first or second immunization) were compared to participants who did not pause their before or after COVID-19 immunization. The variable age was given as a continuous variable and categorized in three categories (< 40 years, 40–65 years,  > 65 years). Medications were grouped in the categories conventional immunosuppressants (methotrexate, azathioprine, leflunomide, mycophenolate mofetil, tacrolimus, everolimus), corticoids (prednisone, hydrocortisone), TNF inhibitors (etanercept, adalimumab, certolizumab, golimumab, infliximab), other biologics (tocilizumab, ustekinumab, vedolizumab, secukinumab, guselkumab) and other immunosuppressants (e.g., hydroxychloroquine, fingolimod, upadacitinib and others). Medical conditions were categorized in the groups rheumatic disease, inflammatory bowel disease, multiple sclerosis, psoriasis, organ transplant, and others. Continuous variables were expressed as means and standard deviations, categorical variables were summarized as numbers and percentages. To compare categorical variables in 2 × 2 contingency tables Fisher’s exact test and for contingency tables exceeding 2 × 2 format Fisher–Freeman–Halton exact test was used. To test for possible confounders, variables were stratified analyzed calculating and comparing odds ratios [OR]. Age, as the only continuous variable, was compared between two groups using the Welch’s t-test. All statistical analysis was performed with SPSS (Version 28.0, IBM, Armonk, NY).

## Results

**Fig. 1:**
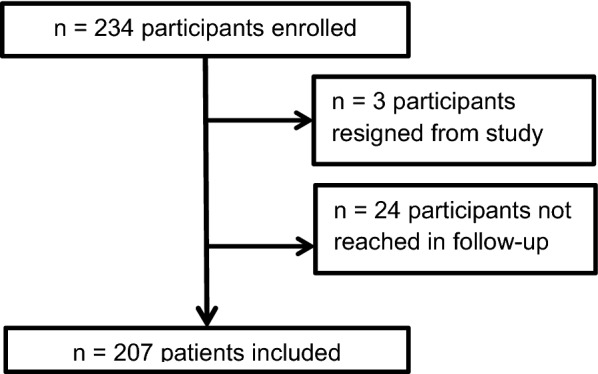
Flowchart of participant inclusion

A total of 234 participants were enrolled in the study, of whom 207 (87.5%) were successfully contacted during follow-up (Fig. 1). Participants were on average 51.9 years old (SD 13.9: 18–86) and predominantly female (70.6%). About one-third (*n* = 75, 36.2%) of the study participants took two or more immunosuppressive medications. The main drug combinations were conventional immunosuppressants plus corticosteroids (*n* = 40 participants) and other immunosuppressants plus corticosteroids (*n* = 16). A total of 305 medications were taken by the enrolled participants, with prednisone (*n* = 63, 20.7%) being the most common, followed by MTX (*n* = 54, 17.7%) and etanercept (*n* = 16, 5.2%).

The most frequent corresponding diagnoses were rheumatic arthritis (*n* = 70, 28.1%), Crohn’s disease (*n* = 26, 10.5%), and psoriasis arthritis (*n *= 21, 8.5%). Those participants who could not be reached during follow-up did not significantly differ from those who were ultimately analyzed in this study—with exception to gender and mean age (see Table 1). Of the 207 participants reached in the follow-up survey, 58 (28.5%) paused their immunosuppressive medication prior and/or after COVID-19 immunization. Seven participants (3.4%) paused their medication only for the first shot, 11 participants (5.3%) only for the second shot and 41 (19.8%) participants for both shots. Most patients reported that they paused their medication for less or equal to two weeks (74.2%).

**Table 1:** Participants’ characteristics compared between analyzed participants and participants lost to follow-up

	Participants included (*N* = 207) *n* (%)	Participants lost to follow-up (*N* = 27) *n* (%)	*p*-value
Gender			
Male	60 (29.4)	2 (7.7)	**0.018**^1^
Female	144 (70.6)	24 (92.3)	
Age, years			
Mean (sd)	51.9 (13.9)	58.6 (14.3)	**0.030**^**3**^
< 40	44 (21.4)	3 (11.1)	0.206^1^
40–65	127 (61.7)	16 (59.3)	
> 65	35 (17.0)	8 (29.6)	
School education			
Lower	16 (8.1)	4 (15.4)	0.203^2^
Middle	59 (29.8)	11 (42.3)	
Upper	121 (61.1)	11 (42.3)	
Other	2 (1.0)	0 (0.0)	
Previous COVID infection			
Yes	3 (1.5)	0 (0.0)	1^1^
First administered vaccine			
BNT162b2 (Pfizer/BioNTech)	131 (67.5)	17 (89.5)	0.138^2^
AZD 1222 (AstraZeneca)	42 (21.6)	2 (10.5)	
mRNA-1,273 (Moderna)	21 (10.8)	0 (0.0)	
Underlying disease^a^			
Organ transplant	14 (6.8)	2 (7.4)	1^1^
Inflammatory bowel disease	38 (18.4)	2 (7.4)	0.185^1^
Rheumatic disease	99 (47.8)	15 (55.6)	0.541^1^
Multiple sclerosis	20 (9.7)	1 (3.7)	0.482^1^
Psoriasis	30 (14.5)	5 (18.5)	0.569^1^
Other	3 (11.1)	29 (14.0)	1^1^
Immunosuppressants^a^			
Conv. immunosuppressants	92 (44.4)	14 (51.9)	0.539^1^
Corticoids	72 (34.8)	10 (37.0)	0.832^1^
TNF inhibitor	39 (18.8)	6 (22.2)	0.613^1^
Other biologicals	39 (18.8)	5 (18.5)	1^1^
Other	51 (24.6)	7 (25.9)	1^1^
Number of taken immunosuppressants			
1	132 (63.8)	15 (55.6)	0.735^2^
2	54 (26.1)	9 (33.3)	
3 or more	21 (10.1)	3 (11.1)	

Data are *n* (%) or mean (SD)

^a^Multiple selection possible

^1^Fisher’s exact test

^2^Fisher–Freeman–Halton exact test

^3^Welch’s *t*-test, bold *p*-values < 0.05

Bivariate analyses showed that participants aged 40–65 were significantly more often pausing their immunosuppressive medication at least for one shot than participants in older or younger age groups (Table 2). Also, females are more likely to pause their immunosuppressive medication than males. These findings were also significant for participants with rheumatic diseases and participants taking conventional immunosuppressants (Table 3). After stratifying for gender the presence of a rheumatic disease was associated only in females significantly regarding the pausing the immunosuppressant therapy (OR 2.17, 95% CI [1.048–4.524], *p* = 0.037). Among men no such association was found (OR 0.91, 95% CI [0.208–3.990], *p* = 0.901.

**Table 2:** Bivariate analysis of sociodemographic and SARS-CoV-2-specific variables between participants regarding their medication pausing status prior to or after first SARS-CoV-2 immunization, school education levels based on secondary education

	Therapy paused (*N* = 59) *n* (%)	Therapy continued (*N* = 148) (%)	*p*-value
Gender			
Male	10 (17.5)	50 (34.0)	**0.026**^**1**^
Female	47 (82.5)	97 (66.0)	
Age, years			
Mean (sd)	52.3 (11.1)	51.8 (14.8)	0.769^3^
< 40	9 (15.3)	35 (23.8)	**0.019**^**2**^
40–65	45 (76.3)	82 (55.8)	
> 65	5 (8.5)	30 (20.4)	
School education			
Lower	6 (10.7)	10 (7.1)	0.689^2^
Middle	15 (26.8)	44 (31.2)	
Upper	36 (62.5)	85 (60.3)	
Other	0 (0)	2 (1.4)	
Previous COVID-19 infection			
Yes	1 (1.7)	2 (1.4)	1^1^
First administered vaccine			
BNT162b2 (Pfizer/BioNTech)	36 (65.5)	95 (68.3)	0.828^2^
AZD 1222 (AstraZeneca)	12 (21.8)	30 (21.8)	
mRNA-1,273 (Moderna)	7 (12.7)	14 (10.1)	
Good Subjective Health status^a^	28 (47.5)	93 (62.8)	0.060^1^
Good Quality of Life^a^	32 (54.2)	92 (62.2)	0.346^1^
PHQ-4D > 5	9 (15.0)	22 (15.3)	1^1^

Data are *n* (%) or mean (SD)

^a^Rated on a 7-point Likert-scale where participants with the highest three ratings are categorized

^1^Fisher’s exact test

^2^Fisher–Freeman–Halton exact test

^3^Welch’s *t*-test, bold *p*-values < 0.05

**Table 3:** Bivariate analysis of immunosuppressant medication and underlying diseases between patients regarding their medication interruption status prior or after first SARS-CoV2 immunization

	Therapy paused (*N* = 59) *n* (% proportion)	Therapy continued (*N* = 148) *n* (% proportion)	*p*-value
Underlying disease^a^			
Organ transplant	1 (1.7)	13 (8.8)	0.073^1^
Inflammatory bowel disease	10 (16.9)	28 (18.9)	0.844^1^
Rheumatic disease	35 (59.3)	64 (43.2)	0.045^1^
Multiple sclerosis	2 (3.4)	18 (12.2)	0.068^1^
Psoriasis	10 (16.9)	20 (13.5)	0.519^1^
Other	6 (10.2)	23 (15.5)	0.380^1^
Immunosuppressants^a^			
Conv. immunosuppressants	36 (61.0)	56 (37.8)	0.003^1^
Corticoids	23 (39.0)	49 (33.1)	0.424^1^
TNF inhibitor	13 (22.0)	26 (17.6)	0.555^1^
Other biologicals	12 (20.3)	27 (18.2)	0.699^1^
Other	9 (15.3)	42 (28.4)	0.051^1^
Number of taken immunosuppressants			
1	32 (54.2)	100 (67.6)	0.072^2^
2	17 (28.8)	37 (25.0)	
3 or more	10 (16.9)	11 (7.4)	

Data are *n* (%)

^a^Multiple selection possible

^1^Fisher’s exact test

^2^Fisher–Freeman–Halton exact test

**Fig. 2:**
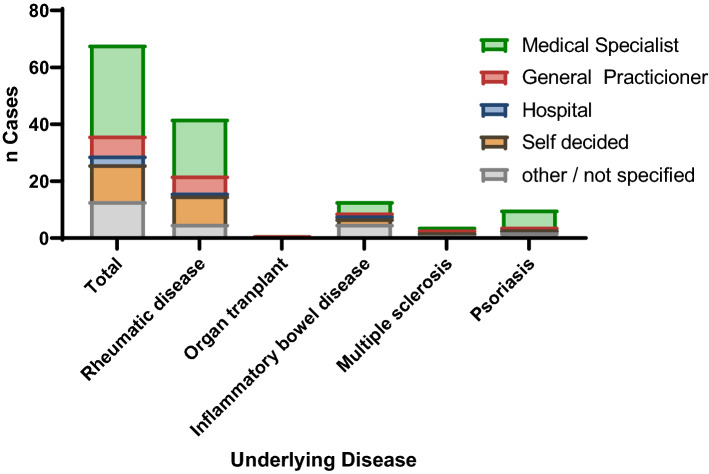
Recommendations to pause immunosuppressive therapy

The majority of participants who paused their immunosuppressive medication (54.2%) reported having received a recommendation to pause immunosuppressive medication from their office-based specialists. Of these participants, 22.0% stated that they had decided to pause the immunosuppressive medication themselves (Fig. 2). Those participants who decided to pause their medication independently without consulting a physician were predominantly female (85%), had rheumatic diseases (60%, Fig. 2), were between 40–65 years old (63.2%), had an upper school education (63.2%) and took mostly conventional immunosuppressants (70%). In comparison participants who paused their immunosuppressive medication based on medical specialist advice showed comparable characteristics to individuals pausing independently but took less often (25%) conventional immunosuppressants.

## Discussion

In our study, 28.5% of the participants paused their immunosuppressive medication during vaccination. Women, patients aged 40–65 years, patients with underlying rheumatic diseases, patients who reported an impaired health status, and patients taking conventional immunosuppressants tended to pause their medication significantly more often. More than half of those who paused immunosuppressive medication said they did so on the recommendation of their specialist. Over twenty percent of participants decided on their own to pause medication. These participants were predominantly female (85%), had rheumatic diseases and a conventional immunosuppressive therapy.

To our knowledge, this study is the first to investigate in a real-life sample which persons pause their immunosuppressive medication prior to COVID-19 vaccination and to investigate upon whose recommendations therapy was paused.

Vaccine hesitancy and reduced vaccine uptake are regular phenomena in immunosuppressed patients and ideas that immunosuppressive therapy is negatively affecting immunization and/or worsening a chronic condition are widespread but often inaccurate [12–14].

Our study did not find any association between pausing immunosuppression and educational level, quality of life, or subjective health status. This finding is in line with systematic reviews on drug adherence among immunosuppressed patients [15, 16]. A recent study among psoriasis patients revealed that 23.6% of patients struggled to adhere to their immunosuppressive medication during the COVID-19 pandemic [17]. This raises the question, of whether the individuals paused immunosuppression in our cohort—and especially those who did so without consulting their provider—are generally less drug adherent.

Data on which immunosuppressive medication in which dosage reduces the immunogenicity of a COVID-19 vaccination relevantly, and whether a temporary interruption of the medication counteracts are still pending [1, 7]. Additionally, there is a lack of studies that would allow a risk–benefit assessment for this decision. Most consensus statements and guidelines suggest continuing medication during COVID-19 vaccination, but some authors discuss that pausing medication might be an option for selected patients, e.g., under rituximab therapy and subsequent full B-cell depletion [18]. However, this would need strategically planning. Contrary, our study suggests, that a considerable number of participants did not involve their provider at all before deciding to pause—although these were often patients with rheumatic diseases and conventional immunosuppressive therapy.

Our study showed, that patients with solid organ transplants are unlikely to pause immunosuppressive therapy. As these participants face the possibility of organ rejection, pausing immunosuppressive medication appears to be a potentially lethal risk. Consensus statements are here rather clear that any interruption should be avoided [8].

But even if providers are involved in the decision-making process, there is still a lack of knowledge about the actual decision-making process that leads to the decision of pausing medication, e.g., who brings the pausing option up and if certain providers are more often tending to suggest a pause than others. Qualitative research might provide here relevant insights about patient-provider interaction as well as beliefs, expectations and experiences with vaccination and immunosuppression of both patients and providers.

This study shows several limitations. Study content including the questionnaires, consent form and the telephone follow-up was only conducted in German language. Participants who indicated pausing medication were asked who advised them to do so . We did not asked participants that did not paused their medication if someone advised them to continue taking their medication. Participants which were lost to follow-up differed in age and gender from the analyzed participants. Due to the fact that we included a real-life sample, not all diseases and medications could be categorized.

## Conclusion

More than a quarter of participants in our study paused their immunosuppressive medication during COVID-19 vaccination and many did so without consulting their treating physicians. Still, evidence is lacking whether pausing medication is associated with a relevant improvement of immune response and thus better protection against SARS-CoV-2. Additionally, participants who pause their medication run the risk of a disease flare-up.

## Data Availability

The datasets generated and analyzed during the current study are not publicly available in accordance with the decision of the involved Research Ethics Boards but are available from the corresponding author on reasonable request within a data sharing agreement.

## Abbreviations

OR: Odds ratio
TNF: Tumor necrosis factor
SARS-CoV-2: Coronavirus disease-2019
SD: Standard deviation
